# Platelets, immune cells and the coagulation cascade; friend or foe of the circulating tumour cell?

**DOI:** 10.1186/s12943-021-01347-1

**Published:** 2021-03-31

**Authors:** Mark P. Ward, Laura E. Kane, Lucy A. Norris, Bashir M. Mohamed, Tanya Kelly, Mark Bates, Andres Clarke, Nathan Brady, Cara M. Martin, Robert D. Brooks, Doug A. Brooks, Stavros Selemidis, Sean Hanniffy, Eric P. Dixon, Sharon A. O’Toole, John J. O’Leary

**Affiliations:** 1 Department of Histopathology and Morbid Anatomy, Trinity College Dublin, Dublin 8, Ireland; 2 Emer Casey Molecular Pathology Research Laboratory, Coombe Women and Infants University Hospital, Dublin 8, Ireland; 3 Trinity St. James’s Cancer Institute, St James’s Hospital, Dublin 8, Ireland; 4 Department of Obstetrics and Gynaecology, Trinity College Dublin, Dublin 8, Ireland; 5 Cancer Research Institute, University of South Australia, 5001 Adelaide, Australia; 6 School of Health and Biomedical Sciences, RMIT University, Victoria 3083 Bundoora, Australia; 7 BD Research Centre Ireland, Limerick, Ireland; 8 BD Technologies and Innovation, Research Triangle Park, NC, USA

## Abstract

Cancer cells that transit from primary tumours into the circulatory system are known as circulating tumour cells (CTCs). These cancer cells have unique phenotypic and genotypic characteristics which allow them to survive within the circulation, subsequently extravasate and metastasise. CTCs have emerged as a useful diagnostic tool using “liquid biopsies” to report on the metastatic potential of cancers. However, CTCs by their nature interact with components of the blood circulatory system on a constant basis, influencing both their physical and morphological characteristics as well as metastatic capabilities. These properties and the associated molecular profile may provide critical diagnostic and prognostic capabilities in the clinic. Platelets interact with CTCs within minutes of their dissemination and are crucial in the formation of the initial metastatic niche. Platelets and coagulation proteins also alter the fate of a CTC by influencing EMT, promoting pro-survival signalling and aiding in evading immune cell destruction. CTCs have the capacity to directly hijack immune cells and utilise them to aid in CTC metastatic seeding processes. The disruption of CTC clusters may also offer a strategy for the treatment of advance staged cancers. Therapeutic disruption of these heterotypical interactions as well as direct CTC targeting hold great promise, especially with the advent of new immunotherapies and personalised medicines. Understanding the molecular role that platelets, immune cells and the coagulation cascade play in CTC biology will allow us to identify and characterise the most clinically relevant CTCs from patients. This will subsequently advance the clinical utility of CTCs in cancer diagnosis/prognosis.

## Introduction

Metastatic progression is the most significant cause of cancer associated morbidity and mortality, causing over 8 million cancer deaths each year [1, 2]. While metastasis is typically viewed as a process that is indicative of advanced stage cancers, recent research suggests that dissemination of tumour cells from the primary malignancy may be an early event in cancer progression [3, 4]. In a clinical setting, there has been limited success in reversing metastatic progression using specific targeting molecules, with the primary barrier being the biological heterogeneity of the cancer cells in the primary and metastatic tumour microenvironment [5, 6]. The process of metastasis is highly complex and understanding the molecular and cellular components involved is critical to our ability to effectively treat cancer, but has proven extremely difficult to define [6, 7].

Metastasis is known to involve several sequential steps referred to as the “metastatic cascade”. This is commonly regarded as the intricate journey a cancer cell must take through different conditions in order to find a suitable distant environment to invade and establish [8, 9]. The ‘seed and soil’ hypothesis put forward in 1889 describes cancer cells as “seeds” which must seek out the appropriate organ microenvironment or ‘soil’ that will support their sustained growth if they are to thrive [10, 11]. This hypothesis still remains a strong argument for the reasoning behind why certain tumour types have a tendency to metastasise to specific organs [5]. Several studies have shown that the distal site acquired for metastatic progression can be determined by specific gene patterns or signatures within the primary tumour, which relate to specific organ sites [12, 13]. The complexity of the tumour microenvironment and cancer cell heterogeneity is further compounded by exposure to the blood circulation system and its physical and cellular components [14–16]. Identifying the molecular mechanisms involved in the initiation of haematogenous metastasis and the interactions with platelets, the coagulation cascade and immune cells could help us better understand specific outcomes in patients with metastatic disease. Thus, the overall aims of this review are to:

1. Outline the contribution of platelets, the coagulation cascade and immune cells on circulating tumour cells (CTCs) in metastasis.
2. Discuss the influence these haematological factors have on CTC biology and the impact on their clinical utility.
3. Define the impact of these heterotypical cell-interactions and discuss potential avenues of targeting CTCs in metastatic disease.

## Circulating tumour cells (CTCs)

**Fig. 1:**
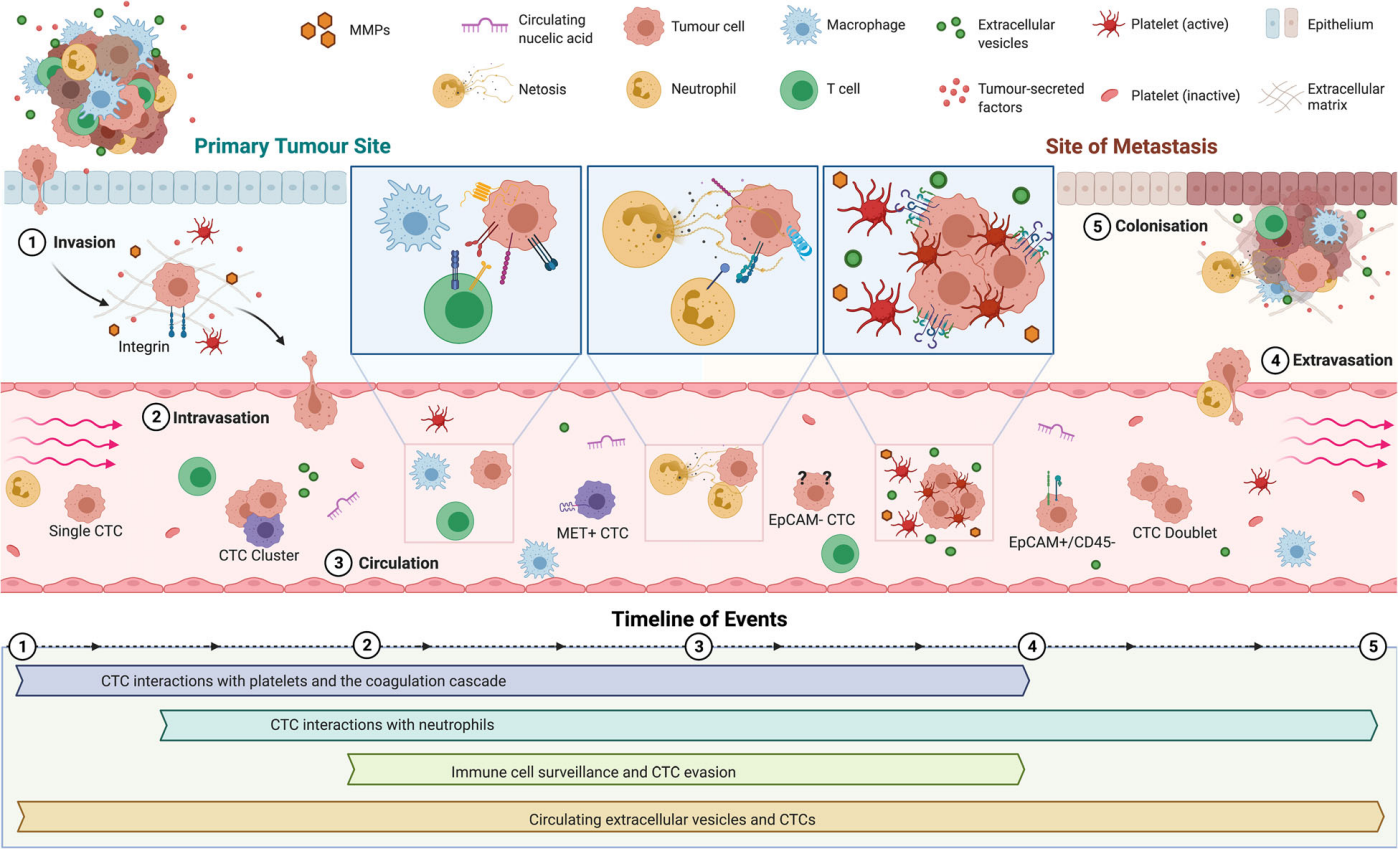
Overview and timeline of CTC-blood interactions during haematogenous dissemination. **1** Invasion: Tumour cells detach from the primary tumour and invade the surrounding tissue. Within the primary tumour, detached CTCs come into contact with platelets and neutrophils within minutes and hours of their dissemination. **2** Intravasation: Degradation of the extracellular matrix and the process of epithelial-to-mesenchymal transition (EMT) resulting from platelet interactions enables the tumour cells to move through the surrounding tissue and finally enter the blood circulation.**3** Circulation: CTCs travel though the circulation. Here, they can exist as single cells, doublets or clusters of CTCs and have been shown to express heterotypical surface receptors, making them difficult to isolate using current technologies. CTCs are constantly interacting with circulating immune cells and other factors in the blood (platelets, circulating nucleic acids, EVs). **4** Extravasation: following the arrival to the site of distal metastasis, mesenchymal-to-epithelial transition (MET) occurs. Platelets aid in the recruitment of neutrophils to metastatic niche. Also, disseminated neutrophil-associated CTCs that arrive have enhanced extravasation capabilities. **5** Colonisation: CTC colonises a secondary site, aided and protected by immune cell-rich microthrombi and host EVs. Here, CTCs and CTC clusters can multiply and eventually develop into a metastatic tumour

The identification and subsequent characterisation of tumour cells that possess distinguishing features, allowing them to leave the primary tumour, journey through the body to a distal organ, and successfully establish a metastatic niche is critical to the understanding of metastatic disease progression [8, 17, 18]. Cancer cells within the circulation are known as circulating tumour cells (CTCs) [19]. CTCs have unique phenotypic and genotypic characteristics, which allow them to survive within the circulation and subsequently extravasate to form a secondary tumour [20]. CTCs have been used as a non-invasive source of cancer cells for the analysis of tumour phenotypes and genotypes (using blood as a so-called liquid biopsy), but their detailed characterisation also holds the key to understanding the biology of blood-borne transition and therefore metastasis. Indeed, the number of CTCs that can be detected at any one time in a patient appears to be relative to the number of “successful” metastatic events. However, effective isolation and accurate enumeration of these cells while they are in the circulation has proven difficult due to the constraints associated with selectively analysing these relatively rare cells. Although cells that disseminate from the primary tumour are usually of epithelial origin, tumour cells can undergo a process known as epithelial-mesenchymal transition (EMT). During EMT cells lose polarity - adherens and tight junctions are dissolved - resulting in a loose epithelial cell that has been dissociated from the epithelial cell sheet [21, 22]. This independent cell differentiates to exhibit several mesenchymal attributes and a more motile and invasive phenotype [21, 23–25]. Within this review we will discuss how platelets promote the initial EMT changes that enables the tumour cells to enter the bloodstream. We will also discuss the sequential interactions between CTCs in the metastatic cascade and cells of the blood circulation, highlighting the complex biology surrounding these putative biomarkers (Fig. 1).

Given that most tumour cells are of epithelial origin, many methods of detection and isolation of CTCs from patient blood samples involve the use of epithelial markers such as pan cytokeratin (panCK) and epithelial cell adhesion molecule (EpCAM) [20, 26–28]. Standard identification and the FDA-approved method of CTC isolation and enumeration from patient blood uses EpCAM positivity and leukocyte common antigen negativity (CD45-) for CTC classification [29]. However, studies have shown that these isolation methods do not fully represent the vast array of CTCs in the circulation. Consequently up to one third of patients with advanced colorectal, breast or prostate cancer do not possess CTCs that meet the standard criteria for cell enumeration [30]. As previously mentioned, the major use for CTCs in the clinic has been in the form of prognostic biomarkers, and their use as biomarkers in many cancers such as metastatic breast cancer and ovarian cancer have been studied extensively [31, 32]. However, despite the prognostic impact of CTC counts that are seen mainly using EpCAM-based capturing methods, this methodology is not capable of detecting the entire, highly heterogenous population of CTCs in patient blood samples. This is due partly to varying EpCAM antigen densities on CTCs as well as potential loss of EpCAM expression following EMT [26, 29, 33]. The process of tumour cell dissemination seen in EMT is often accompanied by a loss or reduction of EpCAM expression on the surface of CTCs, rendering EpCAM-based detection methods unable to capture cells with weak or no EpCAM expression [34–36]. With more recent observations, many have come to the conclusion that a subset of CTCs possess the unique tumour-initiating capabilities or stem cell-like properties that enable them to give rise to a metastatic tumour [17, 20, 25, 37]. Multiple studies have shown that cancer stem cell (CSC) markers are often expressed by CTCs in patient blood samples [25, 32, 38]. The metastatic potential of a tumour can also be based on the presence of a low number of stem cell-like tumour cells found in the tumour tissue [25, 39]. However, no guidelines currently exist for defining a CTC-CSC phenotype, although a common classification of CSCs of high CD44 and low CD24 expression (CD44^+^/CD24^−/low^), is frequently utilised in breast cancer, and breast tumours with this expression tend to exhibit enhanced invasion and metastasis [40]. The variable biomarker expression in CTCs, the capacity to transition between different cellular phenotypes, the detection of single CTCs and clusters of cancer cells, and the detection of CTC and immune cell clusters raises the important question of which hound in the night is the dangerous one?

The classic problems encountered for cancer detection and prognosis apply directly to CTC biology with the search for a “common” biomarker to facilitate efficient isolation, and more specific biomarkers to enable accurate prognosis. Interpatient variability has also been shown to play an important role in the expression of growth factor receptors, adhesion molecules, major histocompatibility complex antigens and proteases [37, 41]. For example, several studies have indicated that the HER2 proto-oncogene defines a particularly aggressive subset of CTCs, that when expressed by the CTCs of breast cancer patients, can indicate a poor prognosis [42, 43]. Similarly, the detection of CK-19 mRNA-positive CTCs in the blood of patients with early stage breast cancer after adjuvant chemotherapy has been shown to be an independent risk factor for chemotherapy-resistant disease [44]. However, the problem associated with CTC surface markers is the heterogeneity of the primary tumours. There are currently no known markers that are universally expressed by all CTCs from a particular tumour type [45]. Despite attempts to standardise criteria, there is conflict regarding the characterisation of CTCs and even more so for immunohistochemical techniques, where reproducibility across various laboratories has been poor [29, 30]. This raises another important point; the CTC field relies heavily on high quality immunochemical reagents which together with robust methodology are essential to assess CTC variability. For example, the non-classical CTCs, such as those lacking EpCAM expression, or possessing the leucocyte marker CD45 are not well understood and defining this biological variability is critical. The full extent of CTC utility as potential clinical biomarkers is currently unknown due to our imperfect methods of isolation and enumeration, as well as our overall lack of understanding of biological differences between homotypic CTCs and heterotypic CTCs. To complicate matters even further, we will now discuss the consequences of CTC interactions with cells from the blood circulation. We will discuss how these interactions influence a CTC’s ability to survive within the circulation and the biological challenges arising from their reciprocal actions.

## Platelets and CTCs

Cancer cell–platelet interactions are a crucial part of cancer metastasis and there may be a physical as well as a biochemical basis for this important biological interaction. However, even though the link has been documented since the late nineteenth century, the interaction and role of platelets in haematogenous metastasis remains largely unknown. Thrombocytosis (excess platelets in the blood) has been linked with a poor prognosis in cancer patients [46]. Platelets are among the first cells of the circulation that CTCs encounter on their journey to metastasise (Fig. 1). CTCs, once disseminated, spend a short time within the circulation before being either trapped within the capillaries they encounter or cleared by patrolling immune cells. Only about 0.1% of single CTCs survive more than 24 h in the bloodstream, with a CTC's half life estimated to be around 1 h, impacting on the cells ability to metastasise [47, 48]. The platelet rich thrombi that is thought to surround CTCs during their initial introduction to the circulatory system offers them physical protection from fluid sheer stresses (FSS) [49]. Platelet cloaking has been shown to protect ovarian cancer cells from FSS in vitro and to increase the production of lactate dehydrogenase, conferring protection to these cells against shear induced damage [50]. At low levels of FSS, thrombin-activated platelets have the capacity to produce a 5-fold increase in endothelial adherence in cervical cancer cells [51]. FSS can also stimulate intravascular survival by upregulation of hexokinase 2 (HK-2) mediated glycolysis in CTCs [52]. The addition of a platelet cloak under these conditions may provide CTCs with a metabolic advantage in circulation, preferentially shunting CTC metabolism to glycolysis. The disruption of platelet-cancer cell interactions could potentially increase shear stress induced destruction of cancer cells, limiting the metabolic advantage and metastatic potential of CTCs in circulation.

Platelets contain within their α-granules, various growth factors that are secreted during platelet activation, such as platelet-derived growth factor (PDGF), vascular endothelial growth factor (VEGF) and transforming growth factor beta (TGF-β) [53]. These growth factors can be utilised by CTCs to aid cell growth [54, 55] and to evade apoptosis when exposed to chemotherapy [56]. Platelets have been found to increase the metastatic potential of solid tumours by inducing EMT through TGF-β signalling [57, 58]. Platelet-derived TGF-β and direct platelet-tumour cell interactions have been shown to harmoniously activate the TGF-β/SMAD and NF-κB pathways in cancer cells, resulting in their transition to an invasive MET phenotype with enhanced metastasis. Inhibition of either TGF-β from platelets or the NF-κB pathway in cancer cells prevented metastasis in vitro [58]. Cancer cell adhesion to, or degranulation of, platelets has also been found to induce pro-survival and pro-angiogenic signalling within cancer cells [59]. The role of platelets in tumour cell proliferation is somewhat more controversial, with platelets manifesting both pro- and anti-proliferative phenotypes [54, 60]. While inhibiting cell proliferation in colorectal cancer cells, platelets promoted metastasis through the release of extracellular vesicles (EVs) that induce EMT and endothelial cell activation [60]. Platelet cargo and release of tumour promoting circulating EVs is an exciting hypothesis that CTCs may utilise within the circulation. Future studies are warranted as to the impact of circulating EVs derived from both platelets and other sources in aiding CTC survival.

The subsequent down-regulation of epithelial marker surface expression through the induction of EMT by platelets may also hide platelet cloaked CTCs from classical antibody based detection [61, 62]. The presence of platelet-derived TGF-β1 in situ in the bloodstream is also crucial for metastasis, as pre-treating tumour cells with platelets from WT mice fails to enhance metastasis formation in mice lacking TGF-β1 in their platelets [58]. Consequently, it is postulated that the platelet cloak provides CTCs with a source of TGF-β1 within the circulation, giving them a more invasive, mesenchymal-like phenotype and extravasation capabilities. Platelets themselves have been found to dictate the formation of early metastatic niches, promoting the recruitment of granulocytes independent of tumour signals through the release of CXCL5/7 chemokines [63]. Blockade of the CXCL5/7 receptor CXCR2, or depletion of either platelets or granulocytes has been shown to prevent the formation of early metastasis in mice. This study also reported that the formation of the platelet induced early metastatic niche occurs within 2 h of tumour cell arrival in the lung vasculature emphasising the near immediate role of platelets in CTC colonisation events. The importance of the CXCL5 axis in CTC metastasis is further exemplified by studies investigating its role in mediating breast cancer metastasis to the bone [64]. Interestingly, RNA sequencing studies for both single cell CTCs and CTC clusters have found gene expression markers of platelets to be overexpressed in their CTC datasets [65, 66]. ITGA2B (integrin alpha-IIb;CD41) and ITGB3 (integrin alpha-V beta 3;CD61) can be expressed by CTCs and are critical for the platelet-cancer cell interaction, as inhibition of ITGB3 prevents the platelet-*tumour* cell interactions [67]. These mRNA signatures may result from platelet vesicle transfer as a consequence of the platelet cloak and could be a putative marker of aggressive CTCs. CTC established cell lines too have been found to express high levels of CXCL5, further elucidating the importance of platelets in fuelling the metastatic potential of CTCs [68].

**Fig. 2:**
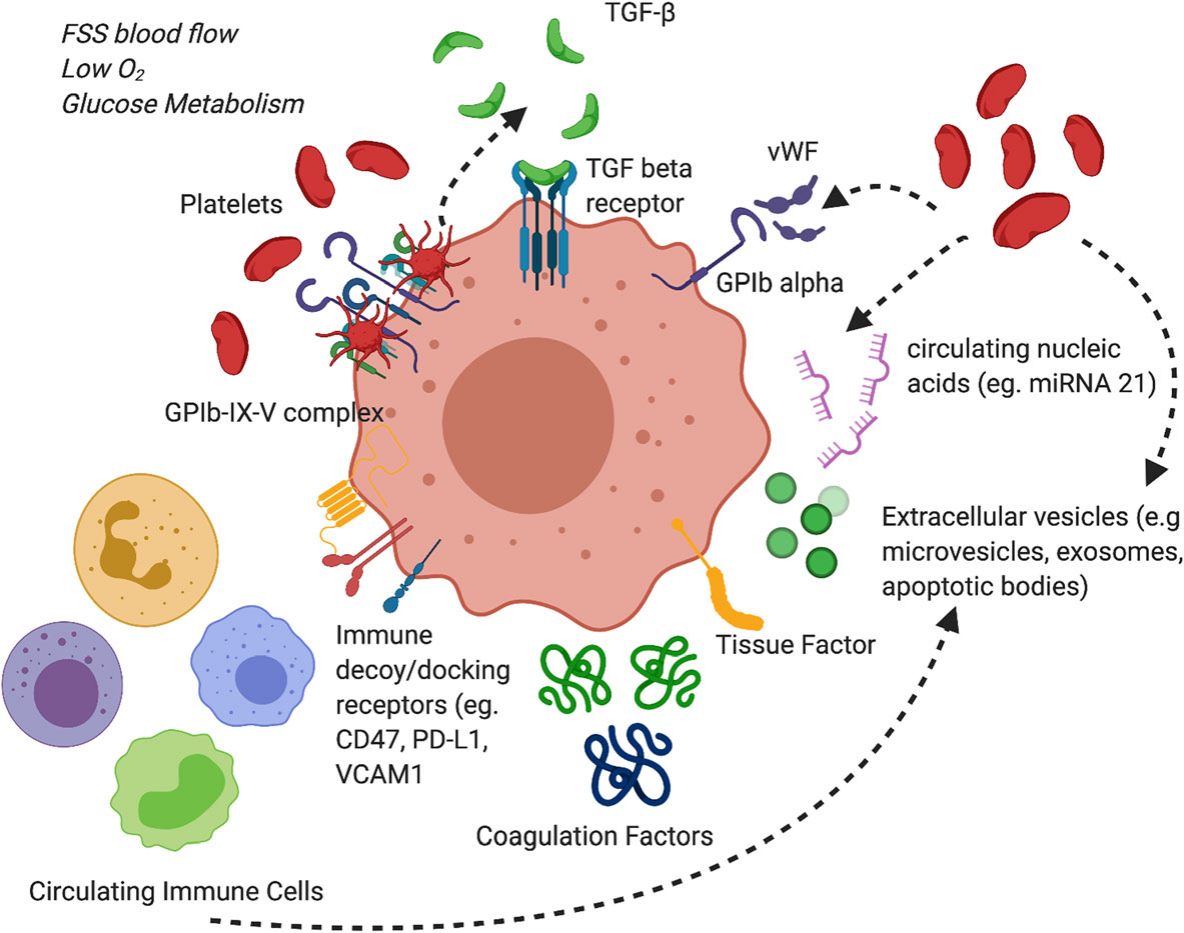
*CTCs interactions with constituents of the blood circulation.* CTCs are exposed to a number of influencing factors while in circulation including fluid sheer stress (FSS), hypoxia, nutrient starvation/glucose metabolism. Platelets, coagulation proteins and immune cells provide either direct or indirect contacts with CTCs to aid in their survival. Platelets are a rich source of TGF-β which promotes EMT. Platelets and coagulation proteins also protect CTCs from FSS through the creation of a rich microthrombi surrounding CTCs. CTCs evade immune detection through the expression of immune decoy receptors such as CD47 and PD-L1. These cells, proteins and circulating nucleic acids/extracellular vesicles can influence not only the phenotype of the CTC in circulation but also its molecular make up and cellular fate within the peripheral blood circulation

Platelet-derived autotaxin (ATX), a secreted enzyme important for generating the lipid signalling molecule lysophosphatidic acid (LPA), interacts with tumour integrin αVβ3 to promote metastasis of breast cancer cells to bone [69]. As ATX is physiologically present in blood, platelet cloaked CTCs may utilise this mechanism for metastasis to the bone in circulation. Interestingly, platelet TGF-β1 and MMP-1 regulate bone metastasis formation, with platelet uptake of tumour-derived proteins aiding in distal metastasis [70]. Platelets can also promote tumour angiogenesis by secreting numerous angiogenic regulators and VEGF [71, 72]. There is a need to better understand the physical and biochemical basis of CTC platelet interactions as they are central to cancer cell survival and metastatic potential (Fig. 2).

## Coagulation cascade and CTCs

Like platelets, proteins of the coagulation cascade are thought to contribute to the protective thrombi formed around CTCs following intravasation to the circulation system. Patients with metastatic cancer have global platelet hyperactivity, which could contribute to the risk of thrombosis [73]. Indeed, venous thromboembolisms (VTEs) are frequent complications in patients with cancer, with the incidence being high in pancreatic, brain, and gynaecological malignancies [74–76]. This hypercoagulable state is due to multiple factors including systemic inflammation and altered expression of circulating blood coagulation proteins such as fibrinogen, Tissue Factor (TF), Factor V (FV), FVII, FVIII, FIX and FX [77, 78]. CTCs themselves have been found to express TF, the known receptor for coagulation factors VIIa and X, which acts as the principle initiator of coagulation [79]. TF plays a key role in aiding thrombin-mediated proteolysis and the formation of tumour cell-associated microthrombi [80, 81]. TF binding with factor VIIa has the potential to facilitate CTC adhesion to endothelial cells as well as the stimulate activation of several inter-cellular signalling pathways (MAPK, PI3K, AKT, mTOR), extracellular matrix remodelling and cell proliferation [82, 83]. TF overexpression in tumour cells has been found to be directly related to the overexpression of mutant oncogenes such as K-RAS and EGFR as well as the loss of tumour suppressor genes p53 and PTEN [84, 85]. TF expression and signalling is implicated in the formation of the metastatic niche and can be upregulated on cancer stem cells [86, 87]. TF can stimulate tumour thrombin production as well as blood coagulation serine proteases such as PARs (protease-activated receptors). The proteolytic activation of these receptors in tumour cells triggers signalling pathways that increase cell migratory/and or invasive abilities through increased secretion of MMPs, as well as the activation and release of soluble proangiogenic factors VEGF and IL-8 [88, 89]. Indeed, FVIIa itself has been found to have a role in tumour pathogenesis, with overexpression increasing the migratory and invasive potential of breast cancer cells through PAR2 activation and the upregulation of β-catenin [90]. Members of the activated protein C pathway, a key anticoagulant pathway, are expressed in gynaecological tumours and also play a role in cancer pathogenesis [91]. These non-coagulation functions of the coagulation cascade postulates a role for these proteins in CTC mediated survival and metastatic potential in the circulation. Procoagulant circulating EVs too may influence CTCs in circulation by facilitating the transfer of proteins and nucleic acids, for instance pro-oncogenic miRNAs to CTCS, including miR-21 and abundant platelet miRNAs [92–94]. The procoagulant nature of the microthrombi utilised by CTCs promotes the recruitment of immune cells such as neutrophils which activate endothelial cells and promote CTC extravasation from the circulation [63]. It is postulated that coagulation proteins promote a hypercoagulable milieu that CTCs utilise on the road to haematogenous metastasis. However, mechanistic studies are required to fully investigate whether expression of these coagulation proteins by CTCs is critical for their dissemination, circulation and colonisation.

As previously mentioned, patients with advanced stage cancers have a high risk of developing a VTE which is thought to be both tumour and treatment related. The presence of CTCs is associated with an increased risk of VTE in breast cancer, with CTC positive patients being 5 times more likely to develop VTE compared to those who are CTC negative [95, 96]. CTC positivity is also linked to plasma D-dimer levels in patients with metastatic breast cancer, further linking CTCs with an increased risk of VTE and hypercoagulation in metastatic cancer patients [97]. However, although increased CTC counts are associated with an increased risk of VTE in cancer, the lethal subpopulation of pro-thrombotic CTCs has yet to be identified, with further experiments and animal model studies being required. While the thrombotic potential and the pathogenesis of coagulation proteins in mesenchymal and CSC biology remains to be elucidated, it is clear that multiple aspects of platelet biology and coagulation proteins are directly involved in CTC survival and metastatic potential.

## Immune cells and CTC interactions

**Fig. 3:**
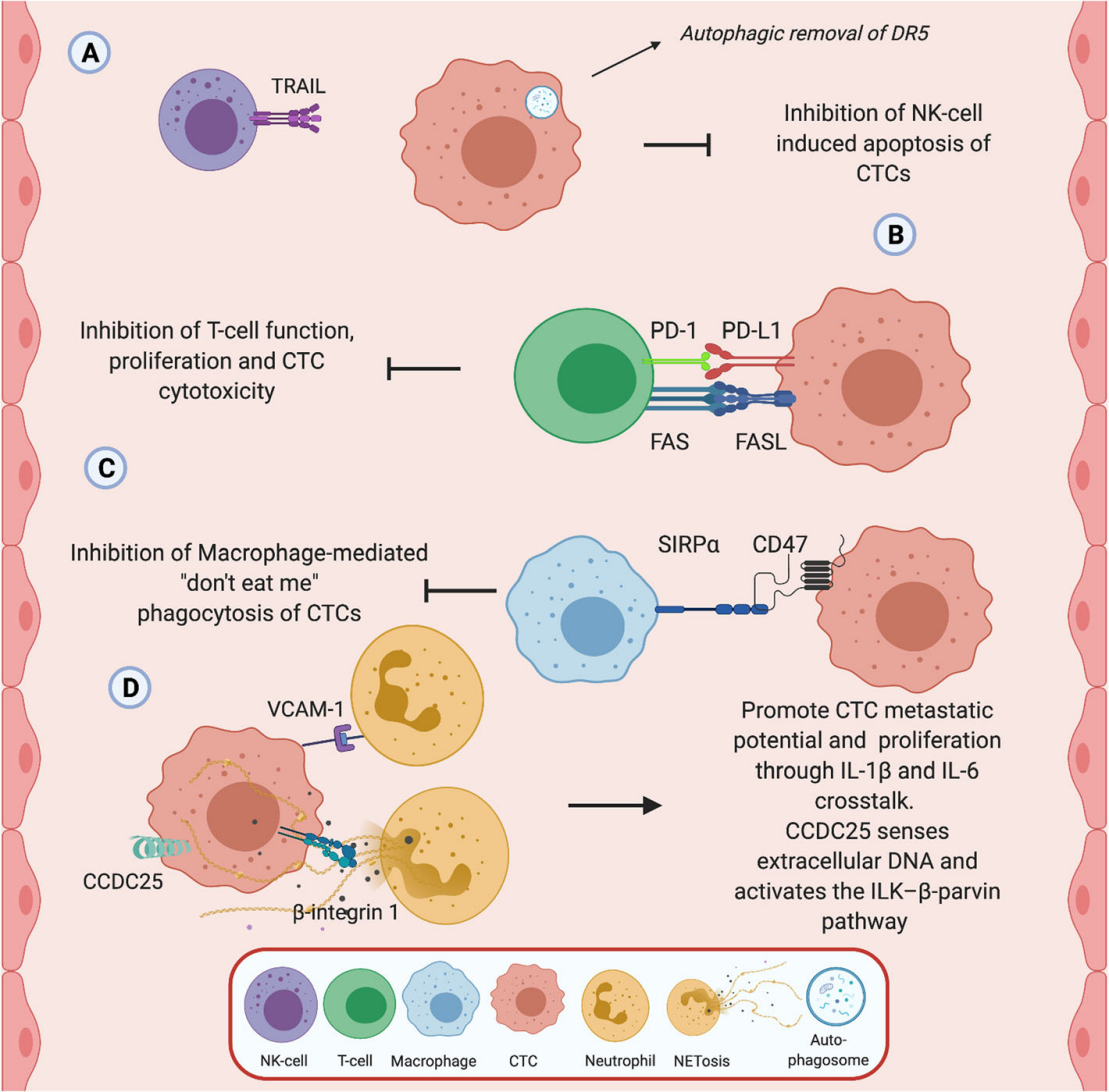
*Interactions between CTCs and immune cells.* CTC interaction with immune cells in the circulation is central to both their survival and ability to form metastatic niches. **a** NK cells, **b** T-cells, **c** macrophages, and **d** neutrophils in the blood circulation have all be found to interact with CTCs. CTCs have shown the ability to resist TRAIL-induced apoptosis via autophagic removal of death receptor 5 (DR5) in vitro*,* thus circumventing cytokine-mediated immune surveillance. CTCs also have been found to express PD-L1 receptor and interact with T-cell PD-1 to reduce anti-CTC T-cell function. Expression of CTC PD-L1 may prevent T-cell mediated cell destruction and offer a potential therapeutic target towards CTCs. Expression of CD47 on CTCs may stimulate “don’t eat me” signals, evading macrophage-mediated phagocytosis and promoting intercellular adhesion and migration of CTCs. Neutrophils, using both direct cell contact and through the production of extracellular traps can promote the metastatic potential of CTCs through increased cellular proliferation. VCAM-1 and β-Integrin1 interactions between CTCs and neutrophils promotes an inflammatory milieu that is conducive for CTC extravasation and formation of the metastatic niche. CTCs too use CCDC25 to sense neutrophil extracellular DNA produced by NETs deposits in organs acting as a chemotactic factor to attract CTCs for distal metastasis

While the immune response to cancer is vast and intricate, involving a range of cell types and molecular mechanisms [98–100], we focus in this review on the specific interactions between immune cells and CTCs in peripheral blood. We also highlight the mechanisms that CTCs use to circumvent tumour immune responses including their ability to hijack specific immune cells (Fig. 3).

Neutrophils are among the first immune cells that disseminated CTCs encounter following entry to the blood circulation. Neutrophils are a subset of mature polymorphonuclear myeloid cells that are first responders to a site of inflammation, and represent one of the body’s first line of defence against pathogens and “foreign” cells [101]. Neutrophils were previously regarded as passive players in the inflammatory response in cancer. However, studies have shown these cells can exhibit both pro-tumour and anti-tumour functions, as a direct result of manipulation from various signals emanating from cancer cells [102, 103]. In metastatic breast cancer, the number of CTCs have been found to correlate with neutrophil to lymphocyte ratios (NLRs), with patients with CTCs and a NLR < 3 having an 8 times greater risk of disease recurrence [104]. The secretion of granulocyte-colony stimulating factor (G-CSF) by tumours recruits circulating neutrophils to the site of a primary tumour [105]. CTCs identified within CTC-neutrophil clusters were also found to express G-CSF and other cytokines involved in neutrophil stimulation, suggesting that recruitment of neutrophils to CTCs may occur rapidly in the circulation or indeed at the primary tumour itself [106]. Tumour killing neutrophils have been found to eliminate cancer cells via the production of hydrogen peroxide (H_2_O_2_) [107]. However, the enhanced presence of neutrophils at the site of a primary tumour has been associated with an overall poor prognosis in a number of cancers [108, 109]. In the context of CTCs, it is speculated that they too are cleared within the circulation using this mechanism but that those cells that do survive, develop resistance to H_2_O_2_-mediated cell death. Indeed, exome sequencing of breast cancer patients positive for CTC-neutrophil clusters revealed a mutation within the TLE1 gene, which may offer some explanation as to why these cells have the ability to overcome neutrophil-mediated killing [106]. Loss of TLE1 results in excessive activation of NF-κB–mediated inflammation in cells and has been shown to aid in cancer progression [110]. Primary tumours with TLE1 mutations too, were also found to have increased levels of neutrophil infiltration and shed significantly more CTC-neutrophil clusters into the bloodstream [106]. The inflammatory milieu created by this mutation creates the perfect opportunity for CTCs to utilise neutrophils for colonisation. The CTC-neutrophil cluster interaction is also mediated through VCAM-1-dependent intercellular junctions based on mouse xenograft experiments. It is also conciliated by a cytokine-receptor crosstalk involving IL-1β and IL-6 [106]. This inflammatory CTC-neutrophil crosstalk also leads to increased proliferation of CTCs within the circulation. Thus, this CTC-neutrophil “piggy back” aids in dissemination, survival and CTC colonisation improving the metastatic capacity of CTCs in the blood of patients.

Neutrophils also have the ability to form an antibacterial trap in the extracellular space to combat bacterial activity which is also of significant importance for CTCs [111]. These neutrophil extracellular traps (NETs) are extrusions of plasma membrane and nuclear material composed of granule components and histones that can bind to and kill pathogens in circulation or within resident tissues [111, 112]. Cell death that occurs as a result of these NETs is NADPH oxidase dependent and is referred to as NETosis [112], with aged neutrophils potentially having a higher potential for releasing NETs compared to non-aged neutrophils. Neutrophils maybe capable of using these NETs to bind to CTCs while in the blood. These CTC-NET interactions are a type of “cloak”, similar to the cloak platelets use to promote CTC adhesion to capillaries and subsequent extravasation at sites of colonisation [113]. NET deposition has been found to increase tumour cell adhesion to the hepatic and pulmonary microvasculature in vivo*,* and in vitro resulting in increased tumour cell migration and invasion [114]. Moreover, NET formation in the absence of systemic inflammation has also found to be sufficient to increase tumour adhesion in vivo. In a murine model, the interaction between CTCs and NETs was found to be mediated through CTC and neutrophil β1-integrin [115]. NETs contain granule components and histones that have been found to act as chemotactic factors to attract cancer cells, rather than just merely acting as a ‘trap’ for them. The presences of NET deposits in the liver or lungs have been found to attract metastasising CTCs [114]. The transmembrane protein CCDC25, which is found on cancer cells, can act as a NET-DNA receptor and senses extracellular DNA to subsequently activate the ILK–β-parvin pathway, enhancing cell motility. Why CTCs were primarily attracted to the liver or lungs by NETs remains an unanswered question, but it is speculated that this maybe in part due to favourable inflammatory conditions created by these organs. Future studies are required as to the potential role of neutrophils and NETosis in aiding colonisation of other distal organs by CTCs, such as the brain.

Circulating monocytes are also myeloid derived immune cells like neutrophils, which can be differentiated into different subpopulations of cells in response to various stimuli, resulting in juxtaposing inflammatory phenotypes [116]. As immature patrolling cells, monocytes can be adapted for clearing cellular debris and to aid in inflammation. Monocytes can also be recruited to the pre-metastatic niche, interacting with tumour cells to prevent their attachment and facilitate their elimination by Natural killer (NK) cells through the secretion of chemokines such as CCL3, CCL4 and CCL5 [117, 118]. CTC counts from metastatic breast, colorectal and prostate cancer were found to inversely correlate with the expression of TLR2 and TLR4 on peripheral monocytes suggesting that these cells play a role in CTC circulation [119]. Macrophages are mature monocytes with phagocytotic potential that can migrate from the bloodstream into tissue. They can be responsible for clearing pathogens and aged/damaged cells like CTCs from the blood stream by phagocytosis [120]. Macrophages detect and destroy altered cells in the blood that are not “self” or which can be regarded as foreign; distinguishing cells that are “self” from those that are not, like CTCs, by identifying “‘don’t eat me” signals on the cell surface of “self-cells”. One of these “‘don’t eat me” signals is CD47, a cell surface glycoprotein utilised by red blood cells, platelets and lymphocytes to protect against their elimination by macrophages [121, 122]. CTCs have been found to express CD47 and utilise it to avoid phagocytosis within the circulation [123]. CD47 binds to the inhibitory immunoreceptor signal regulatory protein alpha (SIRPα) on phagocytotic cells, including macrophages, to induce a signal cascade within the cell that inhibits phagocytosis [123, 124]. As a result, CD47 has been implicated in aiding tumour cell evasion from immune system signals [123]. CD47+ CTCs have also been found to be responsible for tumour relapse and metastasis in patients with breast cancer [125], suggesting that the CTCs may also use this surface marker for colonisation.

While the tumour microenvironment is inherently immunosuppressive [126], cancer progression within the blood circulation is paradoxically associated with an intense immune response. Tumour-infiltrating immune cells, particularly CD8+ T-cells and NK cells, have the potential to restrict tumour outgrowth or reject metastatic tumour cells [127]. Indeed, in most primary tumours, a strong Th1/cytotoxic T-cell infiltration is correlated with increased patient survival [9, 127]. In comparison, the peripheral circulation system is an immunomodulatory complex that is highly reactive to foreign cells such as CTCs. CTCs have been found to express ‘immune decoy receptors’ which aid in CTC T-cell immune evasion within the circulation [128, 129]. Programmed death 1 (PD-1) receptor and its ligand (PD-L1) are important checkpoint proteins for the regulation of the anti-tumour immune response [130]. By binding to PD-1, CTC’s expressing PD-L1 have been shown to limit T-cell function and proliferation to facilitate immune tolerance [130, 131]. However, few studies to date have looked at the exact role of PD-L1 in CTC biology. NK cells are major circulating immune cells that protect the body from a plethora of potentially damaging agents such as virus-infected cells and malignant transformation [132, 133]. NK cells are responsible for marking cells that lack the Major Histocompatibility Class (MHC) 1 marker, which is present on all nucleated cells, to facilitate their destruction by apoptosis [132]. In the early stages of tumorigenesis, NK cells can also detect and eliminate tumour cells [134], but patients where CTCs have been detected, have been found to have reduced NK cell numbers as well as decreased NK cell activity [119, 132]. The exact killing mechanism NK cells use for CTC elimination in the peripheral circulation is not clear, but direct cell-cell contact is thought to be required [135]. NK cells induce tumour cell lysis in the circulation via the secretion of tumour necrosis factor-related apoptosis-inducing ligand (TRAIL), a transmembrane protein of the tumour necrosis factor family [136, 137]. TRAIL expressed on the membrane of NK cells can also bind to a series of death receptors expressed on the cancer cell surface to induce apoptosis [138, 139]. While FSS alone can sensitize CTCs to TRAIL-induced apoptosis [140], in vitro breast cancer models have also shown that CTCs can develop a resistance to TRAIL-induced apoptosis via autophagic removal of death receptor 5 (DR5) [141]. We postulate therefore, that CTCs could potentially be avoiding TNF cytokine-mediated immunosurveillance by downregulation of DR5, which together with protective “platelet cloak” may enable the immunosuppressive nature of CTCs. While the cloaked CTCs are shielded from TNF-α and NK cell cell-mediated cytotoxicity [142, 143], the biochemical basis for this protection may extend beyond the physical barrier provided by the cloak. Platelets can down-regulate NK cell-surface receptor NKG2D via paracrine signalling [144, 145] and actively supress NK cell degranulation and inflammatory cytokine (interferon-γ) production [146], with CTCs potentially benefiting from this mechanism. Platelet-derived VEGF can also supress antigen presentation in mature dendritic cells which in turn limits their immune surveillance capabilities [147]. CTCs can also interact with CD4+ T_reg_ cells and have been associated with defects in T-cell adaptive immunity [148, 149], suggesting that CTCs may actively suppress immune function. Additional functional studies are needed to fully dissect the interaction between CTCs and T-cells to reveal the influence T-cells play in CTC mediated metastasis and to understand the molecular basis of NK cell immune suppression.

## CTC singlets, clusters and survival strategies; safety with friends or potential therapeutic targets?

**Fig. 4:**
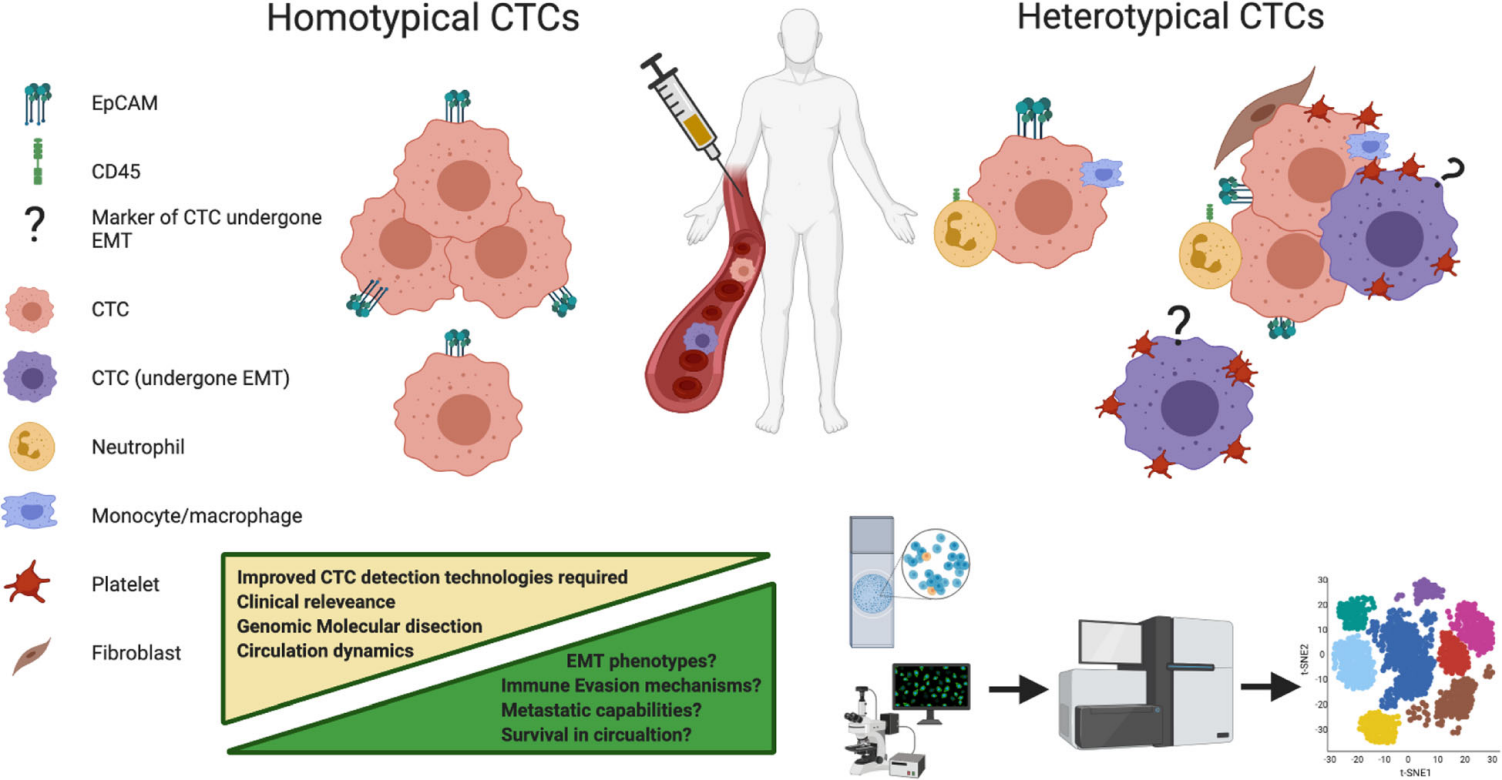
*Homotypical and Heterotypical CTCs and their clinical relevance in malignancies.* For the use of liquid biopsies to reach their full capabilities, CTC isolation technologies are needed to capture the full array of homotypical and heterotypical CTCs that exist in the circulation. Molecular dissection using single cell genomics, transcriptomics and proteomics integration too will reveal the molecular mechanisms that allow for CTCs interactions. This will allow for greater clinical utilisation by identifying the clinically relevant cells and also reveal potential targets for CTC therapeutic interventions. Overcoming these factors must be considered for future CTC enumeration and molecular taxonomy studies

CTCs in the circulation have been shown to exist both as individual cells and as clusters of cells that display homotypic and heterotypic phenotypes depending on their interactions with other cells in the circulation. Although the molecular mechanisms involved in their formation and biology remain largely unknown, CTC-CTC interactions are also of the utmost importance. Clusters of several individual CTCs have been reported in numerous cancers and are indicative of a poor clinical outcome in a number of cancer types [65, 150, 151]. CTC clusters tend to express more mesenchymal markers than epithelial markers, and given that the process of invasion requires a mesenchymal phenotype, this expression profile may confer a selective survival advantage on these cell clusters when in circulation [151, 152]. While it may be obvious, cooperation between similar cell types shielding one another from shear forces, environmental or oxidative stress, and or immune assault in the circulation is advantageous to CTC clusters (Fig. 4). In this context, heterotypic clusters containing more durable stromal or immune cells aggregated with CTCs may provide additional benefit, which in the most simple terms, protect the core of the CTC cluster. Following on, it has been proposed that CTC clusters are less likely to undergo anoikis or apoptosis than a single CTC, advocating that the plakoglobin-dependent intracellular adhesion proteins holding the cells together could confer a survival advantage on the cluster [153].

Understanding single cell CTC and CTC-cluster metabolism and how these cancer cells interact with other cells in circulation to influence changes in physical properties, metabolism and metastatic potential is of the utmost importance. While in the circulation, oxygen deprivation may be even more severely restricted than that of the primary tumour such that only the toughest cells survive, it may nevertheless provide access to nutrients to support the CTCs that promote or require alternate metabolism. Understanding the influence of circulatory cells on single CTCs, and CTC homotypic and heterotypic cell clusters in the rewiring of the cancer cell metabolic network and phenotypic potential is therefore paramount. As cells such as platelets during cloaking are known to alter EMT which itself impacts the expression of genes involved in metabolic pathways, the EMT phenotype of CTC clusters needs to be investigated [154–156] given the strong relationship between cancer cell growth and migratory potential, and their contribution to the metastatic process. While these pathways have been shown to be relevant in single CTCs, differences in cancer cell metabolism associated to CTCs with mesenchymal characteristics or CTC clusters including platelets and /or other immune cells remains to be elucidated. The influence of coagulation factors on CTC metabolism also remains unclear. However, as glucose metabolism has been found to influence coagulation factor expression and is associated with an increase risk in VTEs [157], it is postulated that the metabolism of the procoagulant microthrombi cluster of CTCs is altered and influenced by the expression of coagulation proteins such as TF in the circulation. CTCs may tap into or directly modulate the metabolic function of surrounding cells and this could be a primary weapon in disarming immune functions that are highly glucose dependent. The delineation of potential mechanisms of the metabolic “champion” profiles of metastatic CTCs, and its influence on the colonisation potential of CTC clusters may lead to the development of new therapeutics that seek out and interrupt CTC cluster metabolic pathways that are critical to their survival.

Further molecular dissection of the cells within the CTC heterotypic cluster and the development of standardised markers to detect CTCs that have undergone EMT are necessary to fully dissect the biology of CTC clusters and elucidate any further potential weakness of these lethal CTCs. (Fig. 4). Interestingly, next generation sequencing of homotypic CTC clusters illuminated a potential weaknesses; in a mouse model, Na+/K+ ATPase inhibitors enabled the dissociation of CTC clusters into single cells, leading to DNA methylation remodelling at critical sites and metastasis suppression [66]. Currently a clinical trial (NCT03928210 is underway to investigate the effects of Digitoxin on CTC clusters in breast cancer patients. Digitoxin itself is a well-established and safe Na+/K+ ATPase inhibitor, and if shown efficacious, could be given in combination with standard chemotherapy for metastatic breast cancer. Further observational clinical trials are also under way to interrogate key signalling networks that are active in CTCs clusters (NCT04520672. Data from these trials will better inform us as of potential drug candidates to target CTC clusters. The targeting of heterotypical clusters may prove somewhat more difficult due to the interactions of platelets and immune cells.

Targeting the interactions between platelets/coagulation cascade potentially offers an avenue for CTC disruption. Aspirin is a well-established anti-platelet agent and has shown the ability to disrupt the platelet-cancer cell interaction. Aspirin has the ability to inhibit the release of MMPs from platelets thereby preventing the degradation of the ECM and reducing the invasive potential of CTCs [158]. Previous studies have shown that ovarian cell line models induce high levels of tumour cell induced platelet aggregates (TCIPAs) and therefore may provide a useful model to investigate if aspirin can inhibit ovarian TCIPAs in the initiation of CTC metastasis [158]. Platelet function can also be inhibited by integrin subunit α_IIb_ inhibitors, which have been reported to reduce metastatic cancer progression [159, 160]. Similarly, the experimental blockade or deletion of key platelet receptors on CTCs, such as Glycoprotein Ib-IX-V and Glycoprotein VI, can interfere with the formation of TCIPAs, and significantly decrease their metastatic potential [161]. The inhibition of platelet aggregation in vivo by a highly specific Glycoprotein α-IIb β3 receptor antagonist or aspirin [162] has the potential therefore to impact CTC function and downstream metastasis. Clinical trials such as the “Add-Aspirin” trial (NCT02804815) are currently underway and designed to assess whether regular aspirin use after treatment for an early-stage cancer can prevent recurrence and enhance survival rates in patients [163]. It would be very interesting if CTC monitoring could be included in such a trial, as the use of aspirin may potentially diminish the platelet-rich thrombi associated with the initial stages of metastasis and prevent the onset of metastatic disease. The use of direct oral anticoagulants (DOACs) may also offer a potential avenue for targeting CTCs. However, current data is inconclusive as to their effectiveness at reducing metastatic disease in patients and in vitro studies have so far been disappointing [164].

Targeting CTCs ability to evade immune detection is an exciting avenue in immuno-oncology. The utilisation of PD-L1 by CTCs as a means of immune evasion is a potential therapeutic target for metastatic disease, but is still somewhat in its infancy for solid tumours that undergo haematogenous metastasis. PD-1/PD-L1 inhibitors that are currently in use in the clinic and undergoing clinical trials use CTCs to monitor treatment response [165, 166]. However, to date no trial data exists for the direct targeting of CTCs by immunotherapies. In advanced lung cancer and gastrointestinal cancer there is an association between PD-L1 expression in CTCs and poor prognosis in patients [167, 168]. This association is hypothesised to be a result of PD-L1 positive CTCs that reflect an immunosuppressive tumour microenvironment, which can promote tumour relapse, and PD-L1 positive CTCs with a higher metastatic potential owing to their increased immune evasion capacity [131]. This heterogeneity between primary and metastatic tumours indicates that a single core biopsy could be inefficient at gauging the level of PD-L1 expression at all tumour sites, and CTC expression levels could also be flawed in this way. This comes back to a basic problem in the CTC field where there are limitations with CTC isolation and their characterisation, which is compounded by the need for alternate biomarkers that can effectively report on the complex biology of CTCs, as discussed in this review. It is possible that some metastatic or circulatory event could confer a change of PD-L1 expression on CTCs. Further research is need investigating the altering of PD-L1 profile between the metastatic tumour and CTCs. It is also not known whether PD-L1 expression is altered between single CTCs and that of CTC clusters, or whether the cells that constitute a heterotypical CTC cluster exhibit increased PD-L1 expression. The evaluation of CTC expression in metastatic NSCLC patients that were treated with the PD-1 inhibitor Nivolumab [169], showed the detection of PD-L1 positive CTCs by CellSearch post-treatment correlated with immunotherapy resistance in 14 of 19 patients. In addition to indicating that PD-L1 positive CTCs may be a marker of immune escape [169]. This study also highlights the potential for CTCs as a tool to monitor changes in PD-L1 expression in tumour cells during radiation therapy, which can potentially be prognostic for response to treatment [131]. Further studies are needed to illustrate whether PD-L1 expression in CTCs is acquired at the primary tumour or only while in the circulation, and to elucidate the possible reasoning behind differing PD-L1 expression profiles of CTCs and primary and secondary tumours. Interestingly, combination therapies targeting markers of CTC expression such as HER2/3 or EpCAM in conjunction with immunotherapies may offer an alternative approach to single-agent immunotherapy [170, 171]. The combination of EpAb2–6 (anti-EpCAM monoclonal antibody) with atezolizumab (anti-PD-L1 antibody) has been shown to almost completely eliminate tumours in an orthotopic model of human colorectal cancer. Also, duel targeting of the HER3 receptor using an antibody-drug conjugate, U3–1402, has been found to charge the anti-tumour effects of PD-1 inhibition alone in mice [172]. These combination therapies significantly enhanced anti-tumour immunity and may offer an avenue to prime otherwise immunotherapeutic “cold” tumours. Further studies are warranted to investigate whether dual-targeting of these markers results in altered CTC expression in patients. However, these studies do highlight potential new combination strategies for cancer immunotherapy in patients with EpCAM+ or HER3+ CTCs and opens the avenue for further combination immunotherapies targeting both CTC surface and immune evasion markers. Finally, with the ever expanding field of CAR-T cell immunotherapies, therapies designed to directly target an individual patient’s CTCs maybe soon be on the horizon.

## Conclusion

Circulating tumour cells are critical components of the metastatic cascade that can be isolated from patients by a simple liquid biopsy, and can provide valuable information about the patient’s tumour profile and prognosis. The clinical utility of CTCs is yet to be fully recognised or appreciated, mainly because we only have a limited understanding of CTC biology. The difficulties associated with the isolation and enumeration of CTCs have hindered our ability to progress the technology for CTC characterisation and this has generated conflict within the field regarding appropriate characterisation, and consequently, variable data sets and conclusions. While many methods of CTC detection exist, the invention of a single standardised method to capture the full plethora of CTCs will allow for a more uniform body of research that would greatly aid future studies and clinical practice. However, this may reveal the inherent adaptivity of CTCs and a greater appreciation for the environment that CTCs inhabit and importance of the cells that constitute this environment. Understanding aspects of CTC cluster biology will be important to better comprehend the dynamics of homotypic and heterotypic CTC clusters as well as how this is influenced by the mutational heterogeneity and metabolic adaptability at a critical point in the metastatic cascade. Targeting the cells of the blood circulation to either prevent the formation of CTC clusters or their interactions with single CTCs in the circulation may lead to a significant delay in metastatic potential and thus increased patient survival. Future CTC studies must take into consideration the influence that the cells of the circulation have on CTC imitation, militance and adaptability during the progression to advanced metastatic disease.

## Data Availability

Data sharing not applicable to this article as no datasets were generated or analysed during the current study. All data and information in this review can be found in the reference list.

## Abbreviations

ATX: Autotaxin
CCL: C-C Motif Chemokine Ligand
CD24: Cluster of differentiation 24
CD44: CD44 molecule (Indian blood group)
CD45: Leukocyte common antigen
CD47: Integrin associated protein
CSC: Cancer stem cell
CTC: Circulating tumour tell
DNA: Deoxyribonucleic acid
ECM: Extracellular matrix
EGFR: Epidermal growth factor receptor
EMT: Epithelial-mesenchymal transition
EpCAM: Epithelial cell adhesion molecule
FSS: Fluid shear stress
FV: Coagulation Factor V
FVII: Coagulation Factor VII
FX: Coagulation Factor X
G-CSF: Granulocyte-colony stimulating factor
HER2: Human epidermal growth factor receptor 2
HER3: Human epidermal growth factor receptor 3
HK-2: Hexokinase 2
IL: Interleukin
iNOS: Nitric oxide synthase
ITGA2B: Integrin alpha-IIb; CD41
ITGB3: Integrin alpha-V beta 3; CD61
K-RAS: Kirsten ras oncogene
LPA: Lysophosphatidic acid
MAPK: Mitogen-activated protein kinase
MDSC: Myeloid-derived suppressor cell
MET: Mesenchymal-epithelial transition
MHC: Major Histocompatibility Class
miRNA: microRNA
MMP: Matrix metalloproteinase
mTOR: Mammalian target of rapamycin
NET: Neutrophil extracellular trap
NK cells: Natural killer cells
NKG2D: Natural killer group 2D
NLR: Neutrophil leucocyte ratio
NSCLC: Non-small cell lung cancer
p53: Tumour protein p53
PAI-1: Plasminogen activator inhibitor-1
panCK: pan Cytokeratin
PARs: Protease-activated receptors
PD-1: Programmed death 1
PD-L1: Programmed death ligand 1
PDGF: Platelet-derived growth factor
PI3K: Phosphoinositide 3-kinases
PMN: Polymorphonuclear
PTEN: Phosphatase and tensin homolog
SMAD: Small mothers against decapentaplegic protein 1
T reg: Regulatory T-cells
TCIPA: Tumour cell-induced platelet aggregates
TF: Tissue Factor
TGF-β: Transforming growth factor beta
TLR: Toll-like receptor
TNF: Tumour necrosis factor
TRAIL: Tumour necrosis factor-related apoptosis-inducing ligand
VEGF: Vascular endothelial growth factor
VTE: Venous thromboembolism
